# A Canadian national guideline on the neoadjuvant treatment of invasive breast cancer, including patient assessment, systemic therapy, and local management principles

**DOI:** 10.1007/s10549-022-06522-6

**Published:** 2022-02-28

**Authors:** Sonal Gandhi, Muriel Brackstone, Nicole J. Look Hong, Debjani Grenier, Elysia Donovan, Fang-I. Lu, Mia Skarpathiotakis, Justin Lee, Jean-Francois Boileau, Francisco Perera, Christine Simmons, Anil A. Joy, William T. Tran, The Canadian National Neoadjuvant Breast Cancer Consortium, Ivan Tyono, Althea Van Massop, Shelyna Khalfan

**Affiliations:** 1 Division of Medical Oncology and Department of Medicine, Sunnybrook Health Sciences Centre and University of Toronto, 2075 Bayview Avenue, Toronto, ON M4N 3M5 Canada; 2 Department of Surgery, London Health Sciences Centre and Western University, London, ON Canada; 3 Department of Surgery, Sunnybrook Health Sciences Centre and University of Toronto, Toronto, ON Canada; 4 Section of Hematology and Oncology, University of Manitoba, Winnipeg, MB Canada; 5 Department of Radiation Oncology, Sunnybrook Health Sciences Centre and University of Toronto, Toronto, ON Canada; 6 Department of Anatomic Pathology, Sunnybrook Health Science Centre, and University of Toronto, Toronto, Canada; 7 Department of Radiology, Sunnybrook Health Sciences Centre and University of Toronto, Toronto, Canada; 8 Department of Radiation Oncology, Hamilton Health Sciences Centre and McMaster University, Hamilton, ON Canada; 9 Department of Surgery, Montreal Jewish General Hospital, and McGill University, Montreal, QC Canada; 10 Department of Radiation Oncology, London Health Sciences Centre and Western University, London, ON Canada; 11 Department of Medical Oncology, BC Cancer Agency and the University of British Columbia, Vancouver, BC Canada; 12 Department of Medical Oncology, Cross Cancer Institute, Edmonton, AB Canada

**Keywords:** Breast cancer, Guideline, Neoadjuvant, Consensus

## Abstract

**Purpose:**

The neoadjuvant treatment of breast cancer (NABC) is a rapidly changing area that benefits from guidelines integrating evidence with expert consensus to help direct practice. This can optimize patient outcomes by ensuring the appropriate use of evolving neoadjuvant principles.

**Methods:**

An expert panel formulated evidence-based practice recommendations spanning the entire neoadjuvant breast cancer treatment journey. These were sent for practice-based consensus across Canada using the modified Delphi methodology, through a secure online survey. Final recommendations were graded using the GRADE criteria for guidelines. The evidence was reviewed over the course of guideline development to ensure recommendations remained aligned with current relevant data.

**Results:**

Response rate to the online survey was almost 30%; representation was achieved from various medical specialties from both community and academic centres in various Canadian provinces. Two rounds of consensus were required to achieve 80% or higher consensus on 59 final statements. Five additional statements were added to reflect updated evidence but not sent for consensus.

**Conclusions:**

Key highlights of this comprehensive Canadian guideline on NABC include the use of neoadjuvant therapy for early stage triple negative and HER2 positive breast cancer, with subsequent adjuvant treatments for patients with residual disease. The use of molecular signatures, other targeted adjuvant therapies, and optimal response-based local regional management remain actively evolving areas. Many statements had evolving or limited data but still achieved high consensus, demonstrating the utility of such a guideline in helping to unify practice while further evidence evolves in this important area of breast cancer management.

## Background

Breast cancer is the most commonly diagnosed malignancy globally, with 2.3 million new cases in 2020 [1]. Outcomes have generally improved particularly in higher income nations, including Canada [2]. This is largely attributed to better screening, improved local therapies, and advances in systemic treatment. In addition, a further appreciation of the breast cancer subtypes and associated disparate biology has facilitated several new approaches to multidisciplinary care that have changed the paradigm of breast cancer management [3]. In particular, practice-changing data available in the last several years has resulted in an increased momentum for the pre-operative, or neoadjuvant chemotherapy (NAC) approach to breast cancer treatment [4–8].

A Canadian national consortium for the neoadjuvant treatment of breast cancer (NABC) has existed since 2010. The most recent meeting of this group of national multidisciplinary experts was in May 2019 (Ontario, Canada). This group assembles national multidisciplinary experts in breast cancer to discuss and disseminate emerging evidence-based guidance across the country, and in particular focus on areas that have incomplete evidence and require expert opinion to help direct practice. Various members of this group have previously published meeting reports and one expert consensus guideline, with a significant focus on the utility of NAC for locally advanced breast cancer (LABC) [4–6]. However, over the last few years, research has increasingly demonstrated the important prognostic and predictive implications of treating certain subtypes of early breast cancer (HER2 positive and triple negative) with NAC [3], irrespective of upfront clinical stage or operability. The routine use of NAC for early stage breast cancer that is operable on presentation is a paradigm shift of great importance, with significant therapeutic and resource implications [7, 8]. Rapidly evolving evidence, the paucity of long-term data in some studies, the use of variable patient endpoints, and drug funding disparities within the country, can create some uncertainty in therapeutic approaches, but also opportunities for ongoing clinical trials [9]. Considering all this, there is an ongoing need for expert opinion to help consolidate the approach to NABC patient management. This is paramount to achieving the best possible uniform outcomes for Canadian breast cancer patients, particularly considering the publicly funded healthcare landscape. International breast oncology guidelines often embed the use of NAC within larger documents pertaining to breast cancer management [10]. In addition, some of the NAC recommendations are resource and practice-setting specific; there also remains some debate around the impact of certain research findings on clinical care (example using pathologic complete response rate as a practice-changing endpoint). Finally, most guidelines, including the recently published American Society of Clinical Oncology (ASCO) document, focus on systemic therapy alone [11], and do not include the subsequent implications of systemic therapy on surgical and radiation therapy decision-making. We, therefore, developed a contemporary, evidence-based Canadian National Consensus on the Neoadjuvant Treatment of Breast Cancer, using validated consensus methodology. This is meant to capture the most up-to-date evidence on optimal patient management throughout the entire treatment journey, while aligning multidisciplinary expert opinion with practice-based consensus from clinicians across the country.

**Guideline type:** Evidence Based Consensus.

**Intended users:** Practitioners who treat invasive breast cancer (pathology, radiology, surgery, medical oncology, radiation oncology, and other involved health professions.)

**Applicable resource setting:** Upper middle to high income nations with access to advanced screening, diagnostic, pathologic, surgical, radiation, and systemic therapy options.

## Methods

### Expert guideline panel

An expert guideline steering committee was established at the most recent Canadian National NABC Consortium meeting (May 2019, Ontario, Canada). The committee was comprised of academic and clinical experts in breast cancer management in the following specialties: medical oncology, surgical oncology, radiation oncology, breast radiology and anatomic pathology. All committee experts practice in academic cancer centres for more than 5 years, treat more than 100 unique breast cancer patients per year, and have demonstrated research and academic impact in NABC (peer-reviewed publications, research grants/projects, clinical trials involvement, and/or academic meeting presentations.) Representation from multiple Canadian provinces was sought.

### Systematic evidence review

**Fig. 1:**
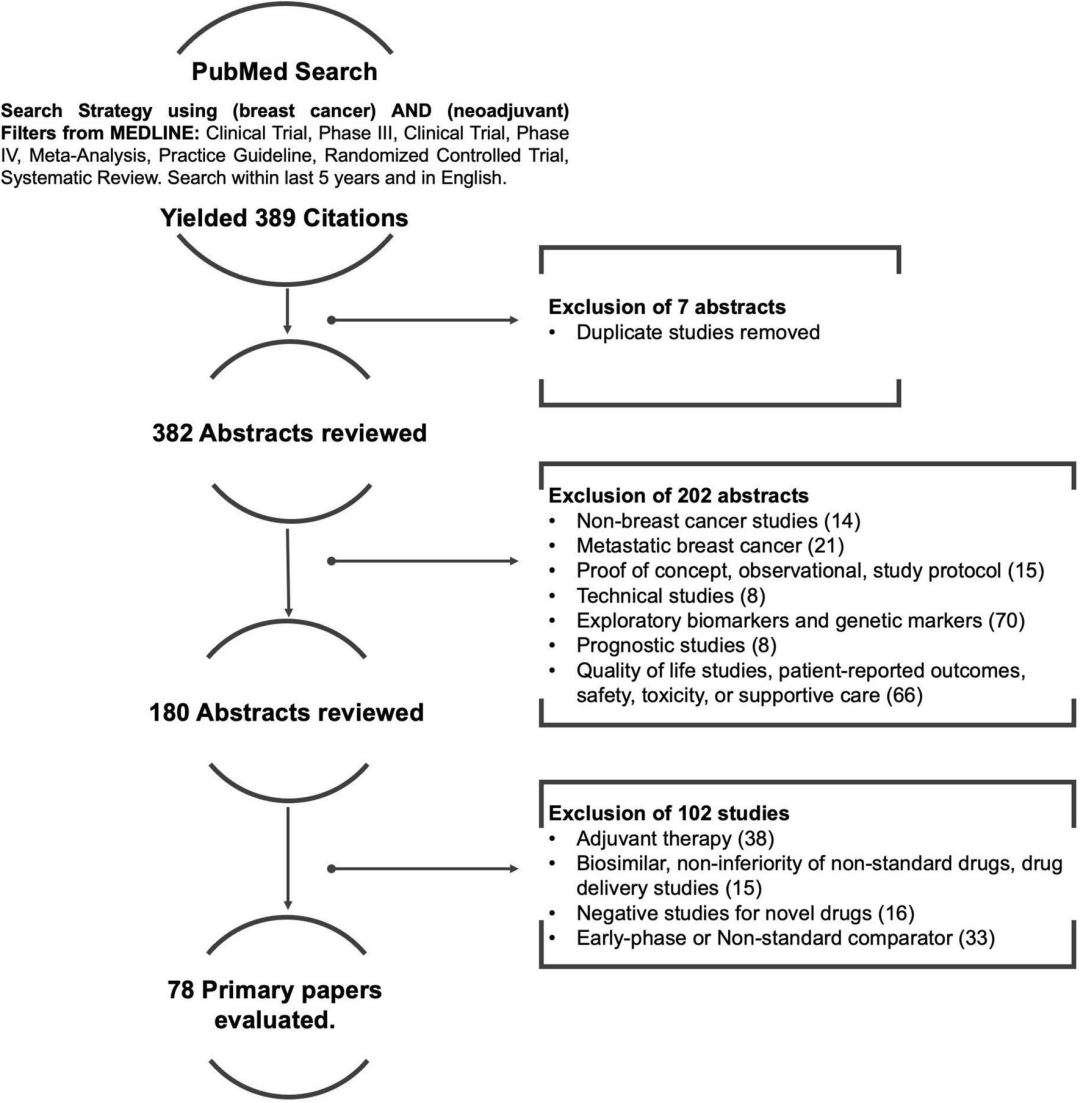
Literature search consort diagram. There were 78 studies included for analysis after applying the inclusion and exclusion criteria

A systematic review of the literature was performed. As management for the neoadjuvant treatment of locally advanced breast cancer and general treatment principles of early breast cancer are well-established [5, 10, 11], the focus of the review was to update established principles of NABC care and highlight areas of new or evolving evidence that in particular would benefit from consensus to help improve practice. The overall focus was defined as the comprehensive management of breast cancer with a neoadjuvant therapy approach, including specific attention to the domains of multi-disciplinary assessment, diagnosis, monitoring, systemic therapy and local treatment. To maintain scope and feasibility, a single database search (PUBMED) was performed with the following parameters: *invasive breast cancer, neoadjuvant, limited to phase 3 or 4 studies, meta-analysis, systematic review, or guidelines published in the English language*. To focus mainly on new developments in this area, the search was limited to the past 5 years (initially October 2015 to October 2020 inclusive); the search was then repeated for November 2020 to May 2021 prior to manuscript preparation, to ensure no new relevant evidence had been published (Fig. 1). In December 2020, July 2021, October 2021, and December 2021, a targeted online gray literature search was completed to review any updated evidence as presented at four high impact oncology meetings (San Antonio Breast Cancer Symposium 2020 and 2021, American Society of Clinical Oncology 2021, European Society of Medical Oncology 2021), and any new published guidelines. The guideline panel decided it was important to capture any relevant new evidence with a select few additional recommendations, to ensure the guideline was most up-to-date. It was decided a priori that if the new evidence did not change the relevance or accuracy of existing recommendations, or greatly change the guideline’s scope or impact, these few select recommendations would not be sent for consensus to prevent delays in final guideline submission.

### Consensus statements and consensus process

The steering committee developed recommendations for consensus based on the evidence review and discussion of important principles of NABC care, as established during and since the last Canadian NABC consensus statement in 2015 [5]. Discussions were held virtually (telephone), and via email correspondence. The statements were further reviewed by five additional clinical experts in breast surgical, radiation, and medical oncology; these expert reviewers were identified from past Canadian NABC Consortium involvement. Representation from multiple provinces was again ensured.

The Modified-Delphi approach is well recognized as a robust consensus methodology, particularly for consensus development in healthcare [12, 13]. This anonymous, survey-based consensus guideline model has several advantages compared to more traditional expert-based or nominal group methods; the latter rely solely on the opinions of a select group of individuals, and can be more subject to bias or the influence of the most vocal members of a guideline committee. Using the modified-Delphi approach, final statements were emailed to potential physician respondents using a secure online survey platform (Survey Monkey Inc., San Mateo, California, USA). Potential respondents were identified as being probable breast cancer clinicians by national or provincial medical society membership, provincial cancer centre affiliation, description of medical practice as available in public domain (example: institutional websites), recommendation by invited colleagues, and/or previous attendance at Canadian National NABC Consortium meetings. Invited participants were instructed only to respond if they had enough clinical expertise and experience in the neoadjuvant treatment of breast cancer to have an opinion. To achieve a broad practice-based consensus on expert recommendations, the guideline committee preferred not to restrict responses by years of practice or number of patients, and assumed respondents would only engage in the survey if they felt comfortable with the subject matter. Invitation to participate in the consensus process was carried out using email addresses for these individuals, as available in the public domain or through personal solicitation from the steering committee, or as shared by invited colleagues. Given the focus of this guideline was an practice-based physician consensus, other health care disciplines, and patient representatives were not included in the statement development or consensus process itself. Widespread physician representation was targeted, including multidisciplinary providers in both academic and community centers and in all Canadian provinces with comprehensive cancer programs.

**Fig. 2:**
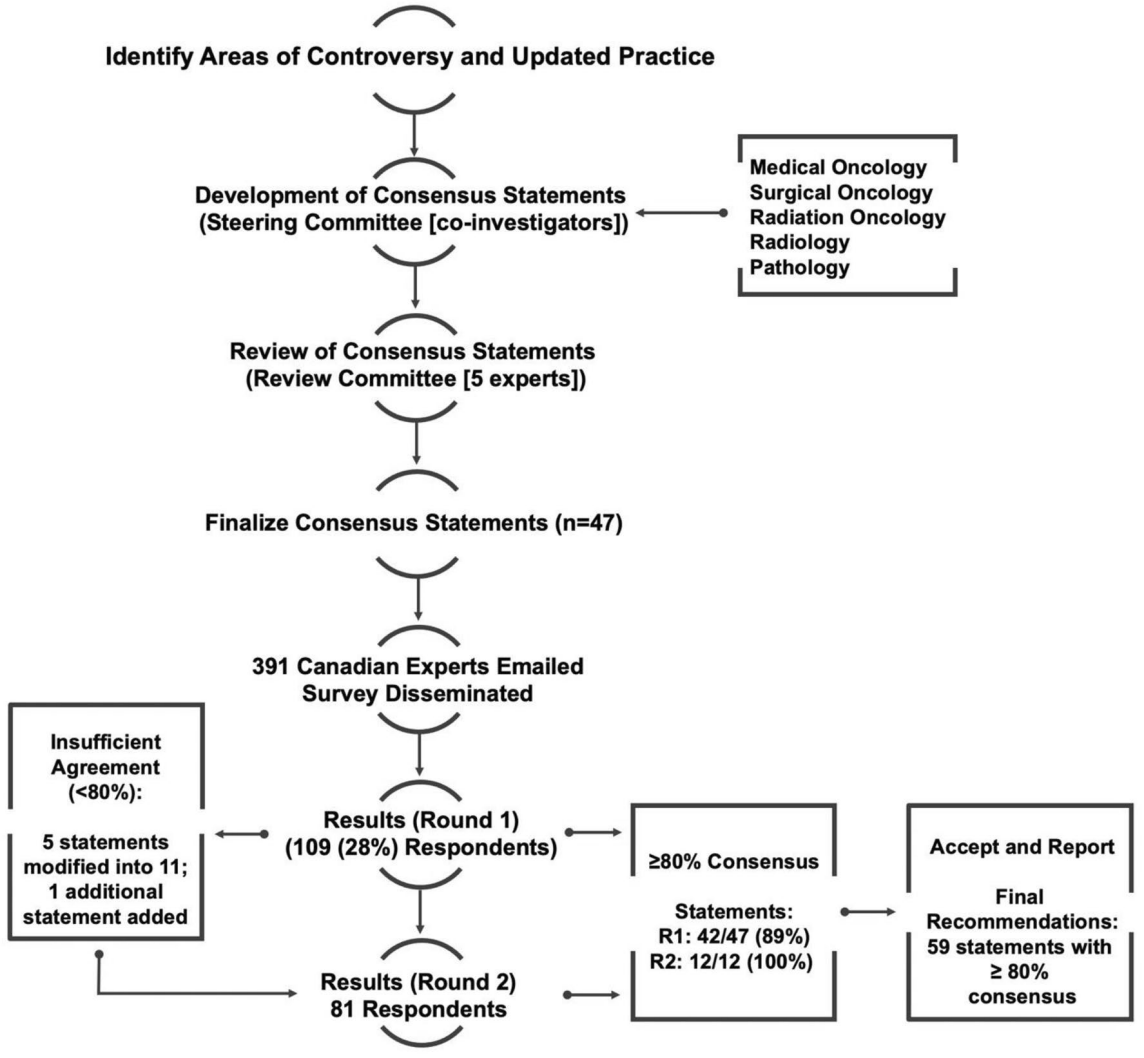
Modified-Delphi Process. Consensus statements were developed by the steering committee based on evidence and relevant discussion. Statements were reviewed by 5 additional experts, finalized, and emailed as a secure online survey. Two rounds were required for consensus of > 79% for all statements

Responses were anonymous; only respondent demographics including discipline, geographic area of practice, and years in practice were collected. Respondents were asked to indicate agreement, disagreement, or neutrality (i.e., "no opinion") to each statement. Given that multiple oncology specialties were involved, respondents were asked to indicate "no opinion" only if the statement was outside of their area of direct practice and not because they had no opinion about a statement related to their specialty. Reminders were sent twice over an 8-week period. Respondents were required to provide detailed qualitative feedback regarding statements they disagreed with. Specifically, respondents were instructed that if they did not agree with a statement in its entirety, to indicate disagreement, and provide detailed feedback regarding the elements they did not agree with. As per Modified-Delphi process, statements that did not achieve consensus were reviewed by the steering committee and modified based on the qualitative feedback as collected by the survey. These statements were emailed for a second round of survey; this was emailed to the same participants. Participants were instructed to respond only if they had responded to the first survey; one reminder was sent over 6 weeks. A third round was planned if required (Fig. 2).

#### Consensus analysis

Agreement statistics were calculated for each statement based on the total number of responses. The denominator for each statement (*N*, Agree + Disagree) was calculated as the sum of respondents who agreed and disagreed. Blank responses and those who indicated "no opinion" were excluded from the total number of responses for each question. The numerator (*n*, Agree) corresponded to the number of respondents who indicated "agree" for each statement. The proportion (*n*/*N*) was converted to a percent value (%) to determine the consensus value. A threshold value was determined a priori; consensus was defined as statements with 80% or more of respondents in agreement; statements with consensus > 79.5% were rounded up to 80%. Statements with less than an 80% (i.e., ≤ 79.5%) agreement level were marked for modification in the next round of survey, as per Modified Delphi methodology. Qualitative feedback was collected from respondents who indicated disagreement with particular statements; this feedback was utilized to modify statements that did not achieve consensus with the initial round.

### Grading of recommendations

The final statements were ranked using the GRADE recommendations for *guidelines *(*Strong or Conditional*) [14], *with consideration of the four domains within the framework for a recommendation’s direction and strength, which include: estimates of effect for desirable and undesirable outcomes of interest, confidence in the estimates of effect, estimates of values and preferences, and resource use* [15]*.* In considering this framework, recommendations were generally considered strong if they were based on positive data and had level 1 or 2 evidence as per the GRADE framework for ranking evidence [14], and met the threshold for consensus. If a recommendation was lacking updated level 1–2 evidence in the last 5 years (acknowledging the review was limited to this timeframe), was *deemed imperative to patient care and received a very high level of consensus (*> *89%), it was also rated as strong.* Recommendations deemed less impactful to patient care, with level 3 or 4 evidence, with preliminary (short term) level 1 or 2 evidence, high resource implications/lack of public funding, or with no published evidence and consensus < 90%, were *marked as conditional.* The term conditional was preferred over “weak” to indicate these statements may have evolving data thatmay strengthen the recommendation over time) and/or the statement may still be impactful for patient care, particularly in certain contexts. Available evidence was linked to recommendations in the “Grading” column (Tables 1 and 2). As the consensus statements may have been based on several sources of evidence with varying strengths, and to illustrate the grading of recommendations was not based on the strength of evidence alone (as discussed above), formal grading of the evidence was not integrated into the guideline table. Finally, additional statements added by the panel (Table 3) were all graded as “conditional” to reflect that they were not sent for consensus.

**Table 1:** Round one consensus

No	Recommendation	*n*/*N*	% Consensus	Grade [references]
*A. General approach*				
A.1	Multidisciplinary assessment, including consultation with the following, (1) Medical oncologist, (2) Surgeon/surgical oncologist and where applicable, plastic surgeon (3) Radiation oncologist. A multidisciplinary tumor board/cancer conference discussion (or access to this forum if required.) Clinical trials/research protocol team, where applicable. Enrollment in clinical trials is encouraged. Nursing and other allied health support as required	105/107	98.13	Strong
*B. Neoadjuvant therapy patient selection*				
B.1	Neoadjuvant chemotherapy (NAC) is the standard of care for all locally advanced breast cancers (LABC), defined as T3/T4 tumors and/or N2-3, and all inflammatory breast cancers, regardless of biomarkers	84/96	87.50	Strong
B.2	There is a suggestion that lobular carcinomas may not respond well to NAC; however, in the absence of level 1 evidence suggesting otherwise (and the potential for mixed histology tumors), LABCs that are lobular will still often be treated with NAC	77/88	87.50	Conditional
B.3	T1-2 tumors with upfront N1 disease that are ER-positive, HER-2 negative and deemed operable, can be considered for upfront surgery in many cases. However, patients may still be offered NAC, particularly to downstage to breast conservation and/or to allow for sentinel-lymph node biopsy	82/96	85.42	Conditional
B.4	NAC is the standard of care for all triple negative and HER-2 positive breast cancer patients, that have T2N0 or TxN1 disease, and are chemotherapy candidates. Tumors should ideally be evaluable for clinical response monitoring (i.e., palpable)	78/93	83.87	Strong [22, 23]
B.5	NAC for triple-negative or HER2-positive tumors that are T1N0 can be considered on a case-by-case basis Specific tumor characteristics (for instance higher grade or T1c lesions) may help better select patients; the main consideration is likely the choice of chemotherapy regimen that may be offered either pre- or post-operatively (for instance, anthracycline sparing for a T1a/b lesion). Please refer to NAC treatment section below	73/86	84.88	Conditional
B.6	NAC can be offered to any patient for tumor down-staging in order to facilitate breast-conservation (this may be determined by tumor-to-breast size ratio, and not necessarily upfront staging)	**72/94**	**76.60**	**Conditional** [24]
*C. Pre-treatment assessment*				
C.1	All patients being considered for neoadjuvant chemotherapy should have pre-treatment breast and axillary imaging, including: Bilateral mammogram: further targeted imaging (i.e., additional mammographic views and ultrasound) of breast and lymph nodes should be performed based on initial findings. Breast magnetic resonance imaging (MRI) can be considered for all patients planned for neoadjuvant therapy (provided MRI-guided biopsy resources are available), especially for lobular carcinomas. Patients should ideally have access to a rapid-diagnostic unit (RDU) or expedited diagnostic examinations, particularly for urgent clinical presentations (example: rapidly growing breast mass, inflammatory breast changes, etc.)	81/95	85.26	Strong [25]
C.2	Biopsy and clips: Core biopsy should be performed of suspicious breast lesions using the most appropriate technique and modality. Fine needle aspiration for suspicious lymph nodes is generally adequate, although core biopsy is appropriate also. For invasive breast tumors that are eligible for breast conserving surgery, a marker clip should be placed to be used for pre-operative localization in the event of complete clinical and radiologic treatment response. Where a sentinel lymph biopsy after neoadjuvant therapy is being considered, a clip should also be placed in a biopsy-confirmed positive lymph node, if applicable	82/94	87.23	Strong
C.3	Staging: Cancer staging by imaging should be performed for all locally advanced breast cancers (T3/4 and/or any positive lymph nodes). This should include computed tomography (CT) of the chest, abdomen and pelvis, and a nuclear bone scan. Positron emission tomography (PET) imaging could also be considered as per local guidelines or research protocols. Patients of any initial clinical stage who have symptoms suggestive of metastatic disease should also receive targeted imaging (symptom directed)	78/92	84.78	Strong
C.4	Pathologic review by an experienced pathologist should be completed on all breast biopsy specimens to confirm an invasive cancer. The pathologist should also report: Histological type (WHO classification), if possible, Nottingham grade, if possible	91/94	96.81	Strong
C.5	Biomarkers should be reported on all core biopsies of invasive cancer, including: Estrogen receptor (ER), Progesterone receptor (PR), Human epidermal growth factor-2 (HER2) receptor status (by IHC and ISH when indicated)	94/97	96.91	Strong
C.6	Sampled lymph nodes should be reported, at minimum, as benign or containing malignant/metastatic cells (designation of breast as primary site is helpful, if possible)	97/98	98.98	Strong
C.7	Lymph nodes with carcinoma cells but no confirmed invasive disease in the breast should be re-sampled with a core biopsy to assess the histology and biomarkers, if applicable. Comprehensive breast imaging, including breast MRI, should be completed in these situations to look for an occult breast carcinoma	92/96	95.83	Strong
C.8	Multi-disciplinary assessment is important for most patients prior to initiation of NAC. Patients should be reviewed by both a medical oncologist and a surgeon prior to finalization of the initial NAC treatment plan. An early radiation oncology assessment should also occur in those patients who may require salvage radiation therapy (example: locally advanced breast cancer and/or inflammatory breast cancer) Multidisciplinary case conference (MCC) assessment should be considered for any patient deemed appropriate by treating physicians	84/97	86.60	Strong
C.9	Patients in whom breast reconstruction may be considered, consultation with a plastic surgeon and radiation oncologist early in treatment planning or initiation is ideal	89/94	94.68	Strong
C.10	A clinical care pathway disseminated to the entire care team can help standardize patient selection and management. In addition, a patient navigator (if resources are available), is often useful to coordinate initial investigations, multi-disciplinary communication, and subsequent patient follow up during key treatment milestones (example: completing chemotherapy, planning for surgery)	83/94	88.30	Strong
C.11	Assessment for fertility preservation (if applicable) should be done prior to the start of neoadjuvant chemotherapy, if applicable and reasonable based on clinical presentation	94/94	100.0	Strong
C.12	Genetics referral, counseling, and testing (if applicable) should be initiated early to permit inclusion of results into surgical planning *Added Note: *Germline mutation testing for appropriate patients may also help select patients for certain adjuvant therapies; please see recommendation G.6 regarding the use of parp inhibitors in BRCA mutation carriers*	92/95	96.84	Strong
*D. Neoadjuvant systemic therapy*				
D.1	Chemotherapy is the standard of care for most invasive breast cancers being treated with pre-operative systemic therapy (neoadjuvant chemotherapy, NAC.) A third-generation chemotherapy regimen including anthracyclines and taxanes should be considered for 6–8 cycles total, in most patients	50/56	89.29	Strong [26]
D.2	Regarding specific NAC approaches: The sequence of agents (anthracycline or taxanes first) can be determined based on patient and disease characteristics, in order to optimize pCR. In general, anthracyclines are often given first. Anthracycline-sparing regimens should generally be reserved for patients at high risk for anthracycline toxicities. Shorter chemotherapy regimens, including taxane-based (such as TC or weekly-paclitaxel with trastuzumab for HER-2 positive) can be considered on a case-by-case basis, considering initial tumor staging (less than 2 cm, N0 disease) and grade (grade 1–2), patient preference, and toxicity considerations. The implications for potentially requiring further treatment post-operatively for residual disease should also be considered, if the initial regimen is not a standard anthracycline-taxane regimen (refer to section on additional adjuvant therapies) Dose-dense (biweekly) regimens are preferred for patients who can tolerate them, particularly for ER-negative cancers, due to the potential for modest improvement in outcomes compared to non-dose dense regimens	**34/47**	**72.34**	**Conditional** [26–33]
D.3	Regarding targeted therapies during NAC: The addition of a platinum to the taxane-containing portion of NAC can be considered for tumors with known BRCA-mutations, or for triple negative breast cancers; this is associated with an increase rate of pCR. The addition of platinums should also be considered if suboptimal or progressive disease is observed in these tumors on the anthracycline portion of NAC. Trastuzumab should be given during the taxane portion of NAC for HER-2 positive breast cancers. Pertuzumab (where accessible) should be considered in addition to trastuzumab, during the taxane portion of NAC for HER-2 positive breast cancers, particularly if there is node positive disease/locally advanced disease upfront. This is to improve the chance of pathologic complete response (pCR rate). NAC with immunotherapy (PD-1 or PDL-1 inhibitors) is considered investigational at this time, and most likely to benefit triple negative breast cancers. Clinical trials can be considered for such patients, if available	**36/48**	**75.0**	**Conditional** [28, 34–51]
D.4	Salvage* therapies for patients that progress on neoadjuvant chemotherapy include immediate surgery, if feasible. Switching to a non-cross resistant chemotherapy, especially platinum based as above for triple-negative cancers or those with known BRCA mutations. This should be followed by surgery, if possible. Radiation, followed by surgery, if possible *Added Note: The term “salvage” refers to a change in treatment meant to address tumour progression on initial therapy	70/75	93.33	Strong [39]
D.5	For patients receiving salvage* radiation (due to progression on NAC, see above), adding weekly cisplatin should be considered as a radiation-sensitizing agent, if the tumor is triple negative, and/or has a known BRCA mutation *Added Note: The term “salvage” refers to a change in treatment meant to address tumour progression on initial therapy	**36/54**	**66.67**	**Conditional**
D.6	Neoadjuvant endocrine therapy is NOT considered standard of care at this time. However, it can be considered for ER-positive, HER-2 negative breast cancer patients with a preference to avoid chemotherapy (due to patient age, co-morbidities or functional status). Chemotherapy candidates that may benefit from neoadjuvant endocrine therapy instead of neoadjuvant chemotherapy have high ER/PR expression and low Ki-67 score at baseline, and/or at the 4- and 12-week mark of therapy, and/or low genomic profiling scores. Research protocols, where appropriate, assessing serial tumor response (such as Ki-67 score) could be considered for these patients. The use of genomic profiling scores to select candidates for neoadjuvant endocrine therapy versus chemotherapy remains investigational at this time. The addition of CDK4/6 inhibitors to neoadjuvant endocrine therapy remains investigational at this time; early evidence suggests it does not improve endocrine-responsiveness or pathologic response rates. The efficacy of neoadjuvant endocrine therapy for down staging most patients with large ER positive tumors to allow for breast conservation, or node-positive patients to (targeted) sentinel lymph node biopsy is currently unknown	52/60	86.67	Conditional [52–64]
*E. Neoadjuvant treatment response monitoring*				
E.1	Standard response monitoring remains by clinical examination at this time. Currently, the use of serial imaging modalities, biomarker analysis, or other novel response monitoring tools remains investigational. Patients should be considered for response monitoring studies where available, particularly where adaptive approaches (changing treatment based on response) can be facilitated, and the patient is a study candidate	75/81	92.59	Strong [25, 65–76]
E.2	Patients demonstrating clinical progression during NAC should have breast and lymph node imaging, ideally utilizing the same imaging modalities as performed pre-treatment	84/88	95.45	Strong [25]
E.3	Patients with documented clinical progression on NAC should have systemic staging (CT and bone scan) to screen for metastatic disease, and access to multidisciplinary discussion	84/85	98.82	Strong
E.4	Imaging to assess post-NAC response for clinical responders should be done on a case-by-case basis, and generally only for those considering breast-conservation, or if deemed useful by the care team for treatment planning. Imaging modalities use for post-NAC assessment should, in general, be the same as initial pre-NAC assessment modalities	79/86	91.86	Strong
*F. Local–regional management after neoadjuvant systemic therapy*				
F.1	Breast conserving surgery (BCS) may be considered for patients with adequate tumor response, in combination with technical feasibility and acceptable cosmesis. In those patients with a hereditary breast cancer mutation, a mastectomy (or bilateral mastectomies) may be the preferred surgical modality, considering patient goals, preferences (including acceptance of chemoprevention), and competing health risks. Multi-disciplinary discussion may be useful in these cases	84/88	95.45	Strong [77–80]
F.2	Patients with extensive calcifications both pre- and post- NAC should be advised about the risks and benefits of surgical removal of all calcifications to ensure resection of possible in situ disease and reduce ambiguity on future surveillance	82/85	96.47	Strong
F.3	The pathologic goal for surgical margins is “no-tumor-on-ink” for resected viable in situ or invasive disease. The presence of residual tumor bed changes at the inked margin should be examined with multidisciplinary discussion to review the utility of any additional local–regional management	78/90	86.67	Conditional
F.4	Patients with initial N1 disease can be considered for (targeted) sentinel lymph node biopsy if: Patients are clinically (by physical exam) node negative prior to definitive surgery AND biopsy proven lymph node was clipped prior to neoadjuvant therapy (if available) and dual tracer is used. At least two sentinel lymph nodes are removed AND pathologic nodal assessment with immunohistochemistry is available AND Patients are appropriately counseled regarding the risk of false negatives with sentinel lymph node biopsy and uncertainty of long-term outcomes	**65/82**	**79.27**	**Conditional** [81–84]
F.5	Axillary lymph node dissection post neoadjuvant chemotherapy should be considered if: Recommended by MCC and/or patients have palpable lymph nodes prior to definitive surgery and/or Initial N2 or N3 disease and/or Have an inflammatory cancer, regardless of chemotherapy response	74/82	90.24	Strong
F.6	The Residual Cancer Burden Index (RCBI) should be incorporated into synoptic pathology reporting of the final surgical breast and lymph node specimens after neoadjuvant therapy, if possible. This is to standardize assessment methods and to provide prognostic information to the clinician	70/77	90.91	Strong
F.7	Endocrine biomarkers (ER/PR) should be repeated on all residual disease specimens where the initial biomarkers were ER negative. HER2 can be repeated on the surgical specimen if there was uncertainty or heterogeneity in HER-2 analysis on initial biopsy	79/85	90.91	Strong
F.8	Clinical factors including stage, age, hormone receptor status, lymphovascular invasion, grade, extracapsular involvement, response to NAC in the primary tumor, and in regional lymph nodes, and initial (clinical) nodal involvement are important considerations for radiation planning following NAC	64/67	92.94	Strong
F.9	Adjuvant breast and regional (lymph node) radiotherapy following breast conserving surgery (BCS): Breast radiation should be offered to all patients following breast conserving surgery. There is currently a lack of evidence regarding the benefit of boost to the tumor cavity post-NAC and BCS. Boost should be considered according to age, grade, positive or close margins, receptor status and extent of residual disease following NAC	55/57	95.52	Strong
F.10	Regional radiation for patients after NAC who have positive lymph nodes on surgical pathology (regardless of upfront nodal status): Patients with 4 or more residual positive lymph nodes at the time of surgery should be offered local–regional radiotherapy Patients with 1–3 residual positive lymph nodes should also be strongly considered for local–regional radiotherapy. There is a lack of clear evidence of the benefit of local–regional radiotherapy for these patients after NAC and BCS	49/57	85.96	Conditional [85]
F.11	Regional radiation for patients with clinically positive nodes prior to NAC: Patients who have cN2-3 at presentation, or multiple high-risk features (age, tumor size, LVI, grade, ER-negative/HER2 positive receptor status, location) should be considered for local–regional radiation following NAC, irrespective of response on surgical pathology. Patients with initial N1 disease should also be considered for local–regional radiation, regardless of response to NAC Those with suspicious but indeterminate N1 disease prior to NAC, and negative nodal dissection at surgery (sentinel or axillary) can be considered for regional radiation on a case-by-case basis	52/54	96.30	Strong [85]
F.12	Treatment of N0 disease after NAC is controversial. Those patients with no residual nodal disease should have a discussion regarding benefits of regional radiotherapy based on presence of high-risk primary tumor features, nodal disease at presentation, and extent of response in lymph nodes and breast to NAC. A comment on fibrous scarring in the lymph nodes on final surgical pathology can be used as a marker of NAC effect, and potential targeting for regional radiation Ongoing trials seek to better define the role of regional RT for N0 disease after NAC, and to assess the benefit of local–regional radiation in patients with in-breast pCR but node-positive disease after NAC	55/58	94.83	Strong
F.13	Post-Mastectomy Radiation Therapy (PMRT): Patients with primary tumors with multiple high-risk features and N0 disease at surgery should also be considered for either local–regional radiotherapy or chest wall radiotherapy. Factors such as primary tumor size, grade, lymphovascular invasion (LVI), age, tumor location and margin status should be considered. Regional/nodal radiation should be considered in patients following mastectomy who have residual nodal disease following NAC: patients with 4 or more residual positive lymph nodes at the time of surgery should be offered local–regional RT. Patients with 1–3 residual positive lymph nodes should also be strongly considered for local–regional radiation. Patients with cN2-3 disease at presentation should be offered local–regional radiation after mastectomy, regardless of response to NAC. Patients with N1 disease at presentation should also be considered for local–regional radiation, regardless of NAC response. Primary tumor characteristics (as above) and extent of NAC response in lymph nodes at surgery should be considered in decision making. There is an absence of clear evidence in this area. Axillary radiation post-mastectomy of N0 disease after NAC is controversial. Those patients with no residual nodal disease should have a discussion regarding PMRT based on the presence of high-risk primary tumor features, nodal disease at presentation, and NAC response in lymph nodes and primary tumor. The presence of fibrous scarring in the lymph nodes on final pathology as a marker for NAC effect in the nodes can be considered (as above)	48/54	88.89	Conditional [86–88]
F.14	Other considerations for radiation treatment selection: Current evidence-based practice is to use conventional fractionation and dose (i.e., 50 Gy/25 fractions). Some institutions may use a hypofractionated regimen (i.e., 40–42.5 Gy/15–16 fractions) within this setting. There is ongoing debate regarding this approach. Ongoing trials seek to evaluate other adjuvant radiation regimens, including after NAC. Sequencing of RT in relation to further adjuvant systemic therapy should be determined based on patient and disease characteristics, and on discussion with the treating medical oncologist In patients who require further breast or axillary surgical management, adjuvant radiation should be initiated once final surgical treatment is complete, and on discussion with the treating surgeon. Stereotactic body radiation (SBRT) for residual in-breast disease in lieu of surgery remains investigational. Currently SBRT remains an option for those patients who are not considered candidates for surgery, in whom metastatic disease is diagnosed prior to surgery, or on clinical trial. Multi-disciplinary discussion is recommended in this setting	42/46	91.30	Strong
*G. Additional adjuvant systemic treatment*				
G.1	Eligible patients who have any residual disease in breast or lymph nodes (RCB I or higher) after neoadjuvant therapy should be offered: Capecitabine for 6–8 cycles for triple negative breast cancer TDM-1 (where accessible) every three weeks for 14 cycles, for HER-2 positive breast cancer. The timing of additional systemic therapy in relation to any further local–regional management will depend on disease risk and phenotype, and patient tolerability of therapies	55/62	88.71	Strong [89–93]
G.2	Timing of adjuvant treatments should be carefully coordinated: Adjuvant TDM-1 for residual HER-2 positive disease can be administered alongside adjuvant radiation, as per the phase 3 clinical trial protocol. Adjuvant capecitabine can be administered before or after local–regional radiation, depending on individual patient and disease characteristics. MCC discussion can be considered for these patients. Decisions regarding the timing of further surgical management (for positive margins or axillary node dissection) in relation to further systemic or radiation therapy should be discussed at multidisciplinary case conference, unless further surgical intervention will help with adjuvant therapy decision-making (for instance: establishing nodal burden for systemic therapy or radiation planning)	59/62	95.16	Strong [89, 94]
G.3	Adjuvant endocrine therapy should be considered for all ER/PR positive cancers as per local practice; endocrine therapy should be maximized in strategy (agents and duration), particularly for high-risk pre-menopausal patients and those with residual disease. This can be given concurrently with adjuvant TDM-1 for eligible patients	73/74	98.65	Strong
G.4	Adjuvant bisphosphonate therapy should be considered for post-menopausal patients (natural or induced menopause)	57/62	91.94	Strong

Recommendations with results in bold did NOT meet the pre-specified threshold for consensus in Round 1 (>79%); these recommendations were modified and sent for a second round of consensus

**Table 2:** Round 2 consensus

No	Revised Recommendation	*n*/*N*	% Consensus	Grade [references]
*Neoadjuvant therapy patient selection*				
B.6-R	NAC can be offered primarily* for tumor down staging, to select patients who are eligible for breast conservation (considering tumor focality, tumor to breast size ratio, and implications for radiation/reconstruction). The likelihood of tumor response based on biomarkers (example: lower chance in ER positive, HER2 negative) should be considered, in addition to the risk of over-treatment with chemotherapy in certain patients *Added Note- Clarification of the term “primary”: in select patients NAC may be offered for down-staging of the tumour as the “primary” goal, however in other patients, NAC is recommended beyond the goal of potentially decreasing clinical tumour burden (example: for HER2 + */triple negative phenotypes*	61/68	91.18	Strong
*Neoadjuvant therapy regimen selection*				
D.2-R.1	Patient and disease characteristics are always considered when choosing NAC. Regarding the specific neoadjuvant chemotherapy regimens: a. The sequence of agents (anthracycline or taxanes first) can be determined based on patient and disease characteristics, in order to optimize pCR. HER-2 directed therapies are generally given with the taxane-component (see targeted agent section)	35/43	81.40	Conditional
D.2-R.2	Patient and disease characteristics are always considered when choosing NAC. Regarding the specific neoadjuvant chemotherapy regimens: b. Anthracycline-sparing regimens can be considered particularly for patients with a high risk for cardiotoxicity. Docetaxel, carboplatin and trastuzumab (TCH) for 6 cycles is a reasonable anthracycline-sparing NAC regimen for HER2-positive disease. *Added note: *It should be considered that the evidence for TCH being equivalent in efficacy to an anthracycline-taxane based neoadjuvant regimen is with the addition of pertuzumab (TCHP); access to pertuzumab is not uniform across Canada at this current time*.	39/40	97.50	Strong 45
D.2-R.3	Patient and disease characteristics are always considered when choosing NAC. Regarding the specific neoadjuvant chemotherapy regimens: c. When using anthracycline/taxane (third generation) regimens, dose-dense (biweekly) regimens may be considered for patients who can tolerate them, particularly for ER-negative cancers (due to modest improvements in outcome.) Tolerability and toxicities should be considered	39/41	95.12	Strong
D.2-R.4	Patient and disease characteristics are always considered when choosing NAC. Regarding the specific neoadjuvant chemotherapy regimens: d. Shorter chemotherapy regimens, including taxane-based (such as TC or weekly-paclitaxel with trastuzumab for HER-2 positive) are sometimes considered on a case-by-case basis, considering initial tumor staging, patient preference, and toxicity considerations. The lack of data in this realm should be noted, as well as the implications for potentially requiring further treatment post-operatively for residual disease, and eligibility criteria for these additional therapies (example: adjuvant TDM-1 data is in HER-2 positive patients with 6 or more cycles of NAC.)Refer to section on additional adjuvant therapies	29/36	80.56	Conditional
D.3-R.1	Pathologic complete response (pCR) has been established as a meaningful prognostic surrogate for particular subtypes of breast cancer, particularly triple negative and ER-negative, HER-2 positive (with the use of anti-HER2 therapy.) Additional systemic therapies improve outcomes for triple negative and HER-2 positive cancers that have residual disease (lack of pCR) after NAC. Therefore, improving pCR rates means less patients with these subtypes may require additional systemic therapy after surgery. Considering these principles; regarding targeted therapies during NAC: a. Trastuzumab should be given during the taxane portion of NAC for HER-2 positive breast cancers	38/39	97.44	Strong [95]
D.3-R.2	Regarding targeted therapies during NAC: b. The evidence at this time shows the addition of Pertuzumab to NAC for HER-2 positive disease does not improve survival outcomes; however, it does improve PCR from NAC when given alongside trastuzumab and a taxane. If accessible, it can be considered to improve pCR (rationale as above). This approach may be preferred for LABC or lymph node positive patients, given the burden of disease, and adjuvant data. However, Pertuzumab is currently not considered standard of care in Canada either for NAC or adjuvant therapy. Access and resource implications should be considered when considering Pertuzumab therapy	35/42	83.33	Conditional [49, 96, 97]
D.3-R.3	Regarding targeted therapies during NAC: c. NAC with immunotherapy (PD-1 or PDL-1 inhibitors) is considered investigational at this time, and most likely to benefit* triple negative breast cancers. Clinical trials should be considered for such patients, if available *Added note: increased pCR rates have been demonstrated with atezolizumab and pembrolizumab in the neoadjuvant setting; pembrolizumab given in the neoadjuvant setting and continued in the adjuvant setting has also most recently demonstrated increased EFS. However, overall survival data is premature. These therapies are currently not standard practice in Canada	54/55	98.18	Strong [98–100, 105, 108]
D.3-R.4	Regarding targeted therapies during NAC: d. There is conflicting evidence regarding the addition of a platinum to the taxane-containing portion of a third-generation NAC regimen; however, if accessible and tolerable, a platinum agent can be considered for triple negative breast cancers, in order to potentially improve PCR. There is evidence that BRCA-associated tumors may not benefit from the addition of a platinum, and therefore ideal patient selection without the knowledge of BRCA-status may be challenging. There is data to support platinums for NAC as an anthracycline-sparing approach. Access remains an issue in many Canadian regions. The addition of a platinum agent to a taxane can also be considered if suboptimal or progressive disease is observed in triple negative tumors during the anthracycline portion of NAC	29/36	80.56	Conditional [101]
D.5-R.1	For patients in whom tumor progression on NAC is treated with radiation, the addition of a radio-sensitizing systemic agent is reasonable to enhance radiotherapy response, with the primary goal of achieving tumor respectability. There is some practice-based data available for the use of weekly platinum agents with radiation for the treatment of triple negative tumors progressing on NAC. This approach can be considered for eligible patients (considering the balance with modestly increased toxicities)	39/49	79.59	Conditional
*Local–regional management after neoadjuvant systemic therapy*				
F.4-R.1	Patients with initial N1 disease can be considered for (targeted) sentinel lymph node biopsy if: Patients are clinically (by physical exam) node negative prior to definitive surgery, AND dual tracer is used, AND At least 3 sentinel lymph nodes are removed. At institutions where lymph nodes are clipped at diagnosis, it is recommended that they are localized at surgery, and excised along with the sentinel nodes. Pathologic nodal assessment with immunohistochemistry should be available. Patients should be counseled that the risk of false negatives is low with a sentinel-lymph node approach that meets the criteria above, but that long term outcomes are still uncertain. Multi-disciplinary discussion with a radiation oncologist prior to finalizing the axillary surgical approach (as with the primary breast tumor) is also encouraged	58/66	87.88	Conditional
*Additional adjuvant systemic treatment*				
G.5	Data regarding the use of adjuvant CDK4/6 inhibitors for high-risk ER positive, HER2 negative patients continues to accumulate. Long term patient outcome data is important before routinely recommending particular agents, including to patients with residual disease after NAC. Clinical trial enrollment in these patients is encouraged	46/47	97.87	Strong [103]

*Added notes were not integrated into the consensus statements, and were included to provide further context and clarity after manuscript review

**Table 3:** Additional statements—not sent for consensus

Neoadjuvant therapy patient selection		% Consensus	Grade [references]
B.7	The use of molecular gene signatures (Mammaprint/Blueprint®, Oncotype DX®, etc.) is well established in the adjuvant setting for ER positive, HER2 negative breast cancers that are lymph node negative and 1–3 node positive. These tools can help select patients that may or may not benefit from cytotoxic chemotherapy in addition to adjuvant endocrine therapy. The utility of these assays in lymph node positive cancers is best established for post-menopausal patients. The pre-operative use of these tools on ER positive, HER2 negative core biopsies remains an evolving area of investigation and practice. Clinicians may consider using these tests, if available, as an approach to better define N0 or N1 ER positive HER2 negative cancers at the time of diagnosis, as high molecular risk that may benefit from NAC, versus lower risk that may benefit from upfront surgery. The impact of clinical nodal status at the time of diagnosis, and menopausal status should be taken into account in these circumstances, as well as consideration that the utility of these tests is currently best established in the adjuvant setting where pathologic stage without the impact of NAC is known. Multi-disciplinary discussion on how to integrate the results of these tests in the pre-operative setting is encouraged (in particular discussion between surgeons and medical oncologists.) It should be noted for clinicians and patients that prospective data validating this approach is still accumulating, and access to these tests for this purpose is currently heterogeneous	N/A	Conditional [55, 60, 106–108]
Neoadjuvant systemic therapy			
D.7	The use of nab-paclitaxel for neoadjuvant therapy in lieu of other taxanes has shown some modest benefit with respect to pCR; however, this is currently not routinely offered or publicly funded in many settings with Canada	N/A	Conditional [102]
Local–regional management			
F.15	Patients with initial N0 disease should always be considered for sentinel node biopsy; this can be done pre or post neoadjuvant therapy	N/A	Conditional [16, 84]
Additional adjuvant systemic treatment			
G.5	Data regarding adjuvant neratanib post neoadjuvant therapy for HER2 positive breast cancer remains preliminary and lacking with respect to sequencing after adjuvant TDM1; patients can be considered for this therapy on an individual basis, ideally in the context of a clinical study	N/A	Conditional [104]
G.6	The use of adjuvant olaparib for triple negative breast cancer with BRCA1/2 mutations has recently demonstrated improvement in patient outcomes but is not yet part of routine practice. Further data continues to evolve. Routine germline mutation testing may be beneficial in appropriate patients to help efficiently identify candidates for this therapy if it becomes routine practice	N/A	Conditional [20]

### Engagement of further stakeholders

The final guideline was reviewed by a pharmacist, breast cancer patient representative, and a neoadjuvant nurse navigator, all affiliated with the Sunnybrook Health Sciences Centre. Their feedback on the premise of the guideline, agreement with recommendations, and on implementation was sought.

## Results

**Fig. 3:**
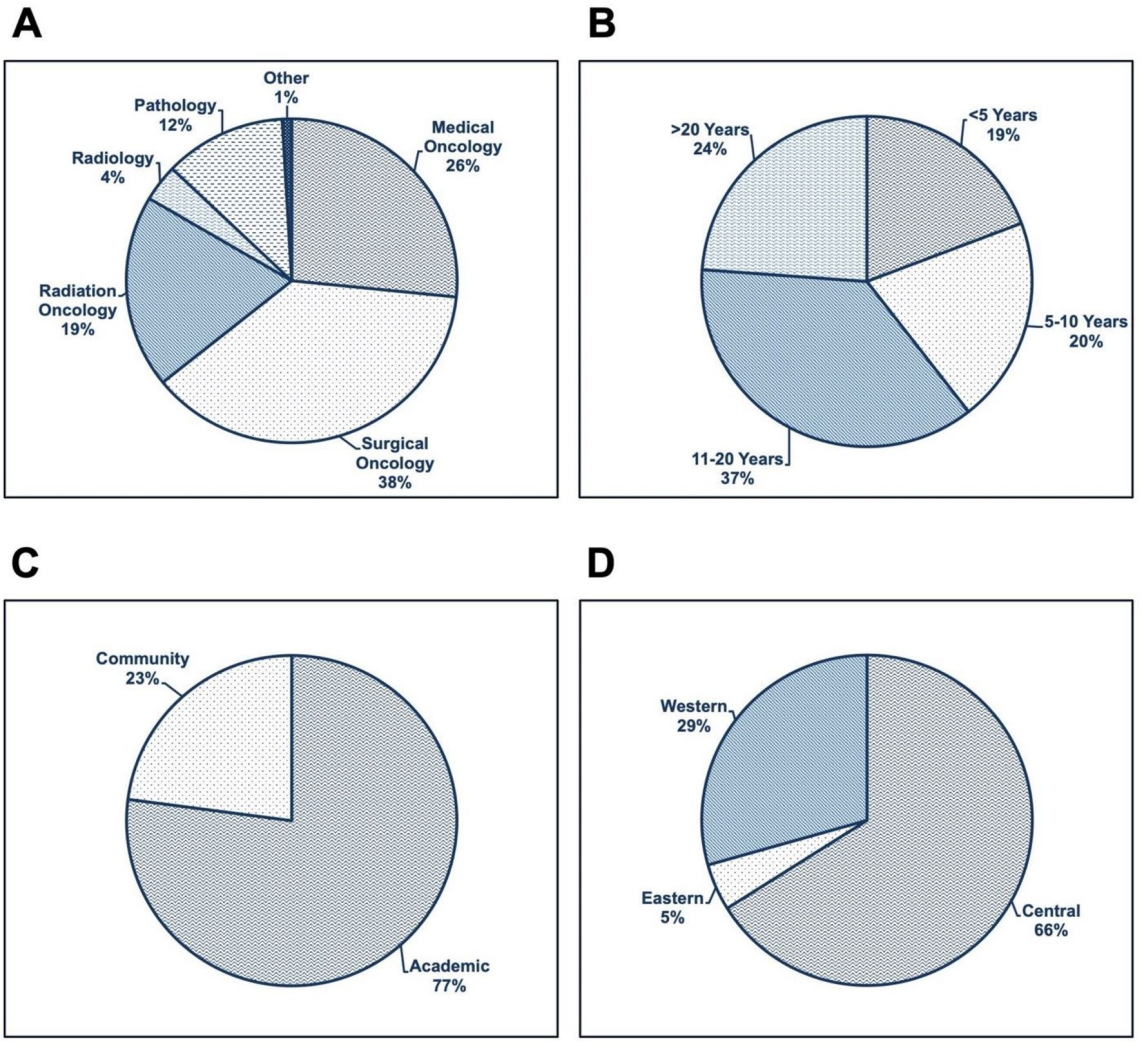
Summary of Expert Respondents' Information. **A** Participants Clinical Specialty. **B** Years of Experience. **C** Practice Setting. **D** Geographic Region

A total of 47 recommendations were initially created by the steering committee and integrated into a consensus survey. Email invitations to complete the survey were sent to 391 clinicians in October 2020. There were 109 participants who completed the survey, for a response rate of 28%. Surgical oncology represented the largest respondent group (41/109; 37.6%), followed by medical oncology (29/109; 26.61%) and radiation oncology (21/109; 19.27%) (Fig. 3). Respondents were predominantly within their mid-career level of practice. Geographical representation was achieved from across several Canadian provinces, although the majority of respondents were located in central Canada (66.0% Ontario and Quebec), and at academic health institutions (77.0%). A summary of all respondent profiles is presented in Fig. 3.

### Consensus agreement: round 1

During the first survey round, 89.4% of questions (42/47) achieved 80% or greater consensus (agreement). A summary of all statements with the levels of consensus is presented in Table 1. Consensus was not reached for five statements under the following domains: patient selection, neoadjuvant systemic therapy, and local–regional management after neoadjuvant systemic therapy. The five statements that did not receive 80% agreement were modified based on qualitative feedback from the survey. These five statements were re-structured into 12 statements and sent for a second round of survey in December 2020. One additional new statement was integrated into the second survey to encompass new data regarding adjuvant therapy that became available as part of the targeted gray literature search in at that time.

### Consensus agreement: round 2

In the second round, there were 81 respondents (81/109; 74.3% of respondents from round 1). A summary of the modified statements in round 2 is outlined in Table 2. All of the modified statements reached ≥ 80% consensus. The new statement on adjuvant therapy achieved consensus. A third round of survey was therefore not required, given the second round achieved complete consensus on this new statement, and all revised statements.

### Systematic review

There were 389 citations found on systematic review; 311 were excluded based on abstract review; criteria are shown in Fig. 1. There were many early phase studies, and those focused on biomarker assessment, a rapidly evolving area of research in the neoadjuvant realm. As much of this data is exploratory or early, these studies were excluded. There were also many studies evaluating imaging response modalities for neoadjuvant therapy; some of these were included and matched to the statement regarding their investigational use. In general, *studies that had negative results or did not meet primary efficacy endpoints, or with early phase data only*, or therapies that had subsequent or conflicting data demonstrating a lack of meaningful impact on patient care, were excluded. The 78 included citations were fully reviewed and matched with guideline statements. For ease of readability and clarity, detailed descriptions of the evidence were not included in the recommendation table itself. Some recommendations did not have associated citations, as they were based on data published before the systematic review timeframe.

### Further evidence review and additional statements

Targeted gray literature review did not demonstrate any impact on the accuracy or relevance of existing consensus statements. However, five additional recommendations were created by the expert guideline panel to reflect important areas of practice deemed not to be captured in the initial or revised statements This included statements on sentinel lymph node biopsy for N0 disease [16], nab-paclitaxel for neoadjuvant therapy [17, 18], and two additional adjuvant therapies, neratinib [19] and olaparib [20]. Finally, the rapidly evolving impact of molecular gene profiling on NABC was decided to be more clearly addressed after final external review. The five additional statements are presented in Table 3. Footnotes were included for a few consensus recommendations to clarify concepts as suggested by external review.

### Additional stakeholder feedback

The nursing, patient, and pharmacist feedback sought demonstrated agreement with the recommendations overall, and in particular with the multi-disciplinary approach to NAC care. Suggestions to disseminate the guideline in patient, nursing, and pharmacy forums were made.

## Discussion

There was a high level of agreement on 59 final statements encompassing the complex, multidisciplinary care pathway of neoadjuvant breast cancer patients. Five additional statements were not sent for consensus but were integrated to reflect the most up-to-date evidence pertaining to NABC at the time of manuscript preparation. Important highlights of this guideline include the recommendation to use neoadjuvant systemic therapy for early (operable) stage HER-2 positive and triple negative breast cancer, and the subsequent use of additional adjuvant therapies for those patients with residual disease after definitive surgery. In addition, this guideline demonstrates the importance of multi-disciplinary collaboration throughout the patient care journey. Finally, this consensus guideline demonstrates a balance between improving patient outcomes in an evidence-based manner while seeking to minimize toxicities, with a focus on individualized decision making, including clinical trial enrollment, particularly where evidence is less robust or still accumulating. This is particularly relevant for local–regional treatment approaches where evidence continues to accumulate from ongoing studies.

### Assessment of resource implications

Given the broad scope of this guideline, including many treatment modalities, formal assessment of cost-effectiveness for individual therapies was outside the scope of this guideline. The committee acknowledged that most of the recommendations were applicable to *High-middle and High income countries*. Due to the robust health technology assessment for cancer drug funding recommendations nationally (pan-Canadian Oncology Drug Review through CADTH) [21], provincially funded systemic therapies for cancer generally have a cost-effectiveness backing within the Canadian healthcare landscape. Therefore, Health Canada approved agents that do not have wide-spread public funding or remain under evaluation (such as pertuzumab) were acknowledged within the recommendations as potentially having resource constraints at this current time. Corresponding statements suggested that accessibility and resources should be considered in particular for these drugs.

### Limitations

Limitations of this guideline include only a 29% response rate to the consensus survey, and potential sampling and non-response bias. Primarily academic physicians responded to the survey, as such, the opinion of breast cancer clinicians in community practice settings may be under-represented. In addition, medical oncologists and surgeons comprised the largest group of respondents, and the opinion of other specialties may not completely be captured. There were also approximately 25% of initial participants who did not respond to the second round of survey, potentially impacting the results. However, this is unlikely, given the high levels of initial consensus on these statements with the first round (range 66–79%). Patient and other health care professionals were not engaged in the initial development of recommendations; their feedback was only sought on the final guideline and implementation plan. Five additional statements were created but not sent for consensus to prevent delay on the timely dissemination of this guideline; however, they are unlikely to impact on the scope and relevance of the guideline in general.

### Summary and knowledge dissemination plan

This work represents an updated Canadian National Consensus on the Neoadjuvant treatment of breast cancer, across all parts of the therapeutic patient journey. A systematic review of recent literature and formal grading of recommendations was also achieved. The evidence was reviewed several times during guideline preparation, ensuring the most updated data was incorporated in a meaningful manner. The neoadjuvant treatment of breast cancer is a rapidly evolving area of clinical and academic interest; data can change quickly and uptake in clinical settings can falter based on sub-optimal knowledge dissemination or hesitancy to change practice. We believe our approach demonstrates Canadian consensus on key areas of neoadjuvant care, integrating available evidence, expert opinion, and practice-based consensus. We believe this guideline can help optimize patient outcomes across the country, by synthesizing the evidence into comprehensive recommendations for clinical care. Furthermore, the presence of national practice guidelines may help to foster clinical and policy change within healthcare organizations and health networks, with the hope of achieving uniformity of practice and thus patient outcomes. Given the importance of ensuring patient management is aligned with best practice, and to help optimize the use of resources and expertise in this area, we hope to achieve broad dissemination of this consensus guideline. A particular strength of this work is the inclusion of all elements of the patient treatment journey, formal grading of recommendations, and also achieving high levels of consensus, particularly in areas where evidence is lacking or evolving. This may help implementation and uptake of practice elements that can standardize Canadian breast cancer care as the neoadjuvant landscape continues to rapidly evolve. This guideline will be disseminated at the next Canadian National NABC Consensus meeting (planned for mid-2022), and ideally at national and international academic forums. There is also much interest in this document from national, provincial and hospital-based cancer programs in Canada to help guide local practice and resource allocation. We hope this guideline will be a strong addition to the published literature in this important area.

## Data Availability

Not applicable.
